# Efficacy of MAO-B and COMT inhibitors on quality of life in patients with Parkinson’s disease: a Bayesian network meta-analysis

**DOI:** 10.3389/fneur.2026.1753555

**Published:** 2026-02-04

**Authors:** Sung Ryul Shim, Yu Jin Jung, Kyum-Yil Kwon, Taeho Greg Rhee, Seon-Min Lee

**Affiliations:** 1 Department of Biomedical Informatics, Konyang University College of Medicine, Daejeon, Republic of Korea; 2 Evidence Based Research Center, Konyang University Hospital, Daejeon, Republic of Korea; 3 Department of Neurology, Kyung Hee University Hospital at Gangdong, College of Medicine, Kyung Hee University, Seoul, Republic of Korea; 4 Department of Neurology, Soonchunhyang University Seoul Hospital, Seoul, Republic of Korea; 5 Department of Psychiatry, Yale University School of Medicine, New Haven, CT, United States; 6 Department of Public Health Sciences, University of Connecticut School of Medicine, Farmington, CT, United States; 7 Department of Neurology, Konyang University College of Medicine, Daejeon, Republic of Korea; 8 Myunggok Medical Research Institute, Konyang University College of Medicine, Daejeon, Republic of Korea

**Keywords:** catechol-o-methyltransferase inhibitors, monoamine oxidase B inhibitors, network meta-analysis, Parkinson’s disease, quality of life

## Abstract

**Background and Objectives:**

Quality of life (QoL) is a critical outcome in the management of Parkinson’s disease (PD), and is often affected more by non-motor symptoms (NMS) than motor features. While monoamine oxidase-B (MAO-B) and catechol-O-methyltransferase (COMT) inhibitors are commonly used with levodopa, their comparative impacts on QoL remains unclear. This study aimed to compare the effects of MAO-B and COMT inhibitors on global and domain-specific QoL in patients with PD using a Bayesian network meta-analysis (NMA).

**Methods:**

A comprehensive literature search was conducted using PubMed/Medline, Cochrane Library and Embase databases from the inception through April 30, 2025. Randomized controlled trials evaluating QoL using PDQ-39 or PDQ-8 in patients treated with MAO-B inhibitors (rasagiline, selegiline, safinamide) or COMT inhibitors (entacapone, opicapone, tolcapone) were included. A Bayesian NMA was performed using the “*gemtc*” package in R. Treatment effects were expressed as standardized mean differences (SMDs) with 95% credible intervals (CrIs). Treatment ranking was estimated using surface under the cumulative ranking curve (SUCRA) values.

**Results:**

Sixteen RCTs comprised of 3,802 patients were included. The combination of extended-release rasagiline and pramipexole (P2B001) showed the most significant improvement in global QoL (SMD = −4.16; 95% CrI: −7.24 to −1.05), followed by rasagiline monotherapy (SMD = −2.38; 95% CrI: −4.32 to −0.42). Safinamide 100 mg significantly improved emotional well-being (SMD = −2.56; 95% CrI: −5.13 to −0.04). SUCRA rankings confirmed the superior probability of benefits for rasagiline-based interventions across multiple QoL dimensions.

**Conclusion:**

This network meta-analysis provides evidence that MAO-B inhibitors, particularly rasagiline and safinamide, may offer broader QoL benefits in patients with PD, especially in NMS such as emotional well-being. These findings support a more symptom-oriented and individualized treatment approach should be provided to patients with PD. Further well-designed head-to-head studies using standardized QoL measures and extended follow-up are needed to confirm these findings and guide clinical practice.

**Systematic review registration:**

Registered in PROSPERO (CRD420251013028): https://www.crd.york.ac.uk/PROSPERO/view/CRD420251013028

## 1 Introduction

Parkinson’s disease (PD) is a progressive neurodegenerative disorder that manifests with a wide range of motor and non-motor symptoms (NMSs), each of which contributes substantially to disease burden and reduced quality of life (QoL) (1, 2). While motor symptoms, such as bradykinesia, rigidity and tremor, remain essential for clinical diagnosis of PD, an increasing attention highlights the Critical impact of NMSs, including cognitive impairment, neuropsychiatric symptoms, sleep disturbances, sensory symptoms and autonomic dysfunctions on long-term disability and QoL in patients with PD (3, 4).

QoL is a multidimensional concept reflecting an individual’s subjective perception of their physical, psychological, and social functioning, autonomy, and environmental context (3, 5). In patients with PD, QoL is significantly impaired not only by motor symptoms, but also often more profoundly by NMSs. Among these, depression and cognitive impairment are consistently reported as robust independent predictors of reduced QoL, frequently surpassing motor severity or disease stage (5, 6). Consequently, QoL has emerged as a CrItical clinical outcome, and there is growing recognition that the ultimate goal of management in PD should be to improve QoL rather than solely focusing on controlling symptoms (7).

Levodopa remains the most effective first-line therapy for managing motor symptoms in PD. However, its prolonged use is often complicated by motor complications such as wearing-off and dyskinesia. As a result, adjunctive therapies targeting dopamine metabolism have become standard practice. Two widely used classes of adjunctive medications include: (1) monoamine oxidase-B (MAO-B) inhibitors (e.g., selegiline, rasagiline, and safinamide) and (2) catechol-O-methyltransferase (COMT) inhibitors (e.g., tolcapone, entacapone, and opicapone), which augment dopaminergic transmission through central inhibition of dopamine metabolism and peripheral inhibition of levodopa degradation, respectively (7–9). Recent pharmacokinetic advances have led to the development of newer agents with once-daily dosing and improved tolerability profiles, such as safinamide and opicapone, thereby expanding therapeutic options. Notably, some of these agents may also provide potential benefits beyond controlling for motor symptoms, with emerging evidence suggesting improvement in NMS and specific domains of QoL, particularly emotional well-being (10–12).

Existing studies assessing the impact of MAO-B and COMT inhibitors on QoL have mainly relied on patient-reported measures such as the Parkinson’s Disease Questionnaire-39 (PDQ-39) and the EuroQol-5 Dimension (EQ-5D). Recent randomized trial–based meta-analyzes indicate that MAO-B inhibitors, when used as adjuncts to levodopa, are associated with modest but statistically significant improvements in global QoL, with relatively consistent benefits in domains such as mobility, activities of daily living, emotional well-being, and bodily discomfort, whereas cognition and social support show limited change. In contrast, COMT inhibitors demonstrate clear efficacy in reducing OFF time, but their effects on QoL indices are less consistent, particularly in long-term or patient-reported outcomes (13, 14).

In this context, while dopamine receptor agonists represent a key component of PD pharmacotherapy and have been extensively studied with respect to both motor and non-motor outcomes, their effects on QoL have already been well characterized in previous randomized trials and meta-analyzes. In contrast, comparatively less attention has been directed toward the QoL effects of dopamine metabolism–modulating agents, including MAO-B and COMT inhibitors.

Regarding potential differences between Western and Asian populations, direct head-to-head randomized comparisons focusing on QoL outcomes are scarce. Although subgroup analyzes and Asia-focused studies, particularly with safinamide, suggest broadly comparable improvements in OFF time and selected QoL domains across regions, the available evidence remains indirect and limited. Therefore, whether treatment responses and QoL benefits differ meaningfully between Western and Asian patients remains unclear (15, 16).

Despite their clinical relevance, most randomized controlled trials (RCTs) have primarily focused on motor-related outcomes, and high-quality evidence regarding their effects on QoL remains limited. Existing data are largely derived from *post hoc* analyzes or observational studies, limiting confidence in comparative effectiveness. Consequently, the comparative impacts of MAO-B and COMT inhibitors on global and domain-specific QoL remains unclear. We conducted a network meta-analysis (NMA) to examine the impacts of MAO-B and COMT inhibitors on QoL-related outcomes in patients with PD. By combining direct and indirect evidence across multiple agents, this NMA allows a comprehensive comparison of the impacts of MAO-B and COMT inhibitors on both global and domain-specific QoL. The ultimate objective of this study is to provide clinically meaningful, comparative evidence that can support more personalized and symptom-based treatment decisions aimed at improving the QoL in patients with PD.

## 2 Materials and methods

This NMA study was registered in the PROSPERO database (registration number: CRD420251013028), and was conducted in accordance with the Preferred Reporting Items for Systematic Review (PRISMA) and Meta-Analyzes guidelines, including extensions for network meta-analyzes (17).

### 2.1 Data sources and literature search

A comprehensive search was performed using Medical Subject Headings (MeSH) terms and text keywords associated with improvement in QoL among patients with PD treated with MAO-B inhibitors and COMT inhibitors. We used the following databases: PubMed/Medline, Embase and Cochrane Library from the inception through the end of April 30, 2025. Boolean operators (e.g., AND, OR, and NOT) were used to include all related search terms. The literature search did not place any restrictions on languages or study designs. Two researchers (SRS and S-ML) manually searched for all relevant studies conducted in clinical trial registries and Google Scholar and independently screened all records. Full search strategies are listed in Supplementary Table S1.

### 2.2 Study selection

Study inclusion Criteria were as follows: (1) patients with a clinical diagnosis of PD, as defined by the individual study protocols, enrolled in RCTs; (2) interventions included the administration of either MAO-B inhibitors (such as rasagiline, selegiline, safinamide) or COMT inhibitors (such as entacapone, opicapone and tolcapone); (3) comparators consisted of patients receiving placebo or active intervention; (4) QoL as outcome measures using validated, PD-specific instruments, specifically the 39-item Parkinson’s Disease Questionnaire (PDQ-39) or its abbreviated version (i.e., PDQ-8).

The PDQ-39 is a disease-specific instrument comprising 39 items across eight domains, providing a comprehensive assessment of health-related QoL in patients with PD. The PDQ-8 is a validated short-form version derived from the PDQ-39, consisting of one representative item from each domain, and was developed to reduce respondent burden while preserving sensitivity to overall QoL changes. Both instruments generate standardized summary index scores, with higher scores indicating worse QoL (18).

To ensure data accuracy and relevance, only peer-reviewed, full-text RCT publications in English were considered. Conference abstracts, case reports, review articles, and non-original research (e.g., editorials and commentaries) were excluded. Two independent investigators (SRS and S-ML) screened all records and extracted data using a standardized form. Final study inclusion was determined through consensus, with cross-checking to avoid data duplicates.

### 2.3 Data extraction

Data were extracted using a pre-defined standardized form and included study characteristics (e.g., author, publication year, country, and design), patient demographics (e.g., sample size, mean age, sex distribution), intervention details (e.g., generic name and dosing information), follow-up period, and outcome measures. For QoL, summary index scores were used in the main analysis, while sub-domain-specific scores were also collected for domain-specific comparisons (secondary analysis). In our main analyzes, only the standard therapeutic dose for each drug [i.e., rasagiline 1 mg, safinamide 100 mg, entacapone 200 mg, opicapone 50 mg and the fixed-dose combination of rasagiline Extended-release (ER) 0.75 mg with pramipexole ER 0.6 mg (P2B001)] was included. In our secondary analyzes, both standard and dose-specific data were extracted to enable dose-stratified comparisons. Only studies reporting sufficient quantitative data were included in the final meta-analyzes.

### 2.4 Network meta-analysis assessment of outcome findings and statistical analysis

A Bayesian NMA was performed using the “*gemtc*” package in R (version 4.3.1; R Foundation for Statistical Computing, Vienna, Austria) (19). To compare seven interventions, we conducted simulations using Markov Chain Monte Carlo (MCMC) methods, incorporating prior distributions and probability models. Convergence of the posterior distributions was evaluated through trace plots, density plots, and the MCMC standard error, and the optimal model was selected accordingly. Posterior estimates of the treatment effect sizes were subsequently derived. To assess consistency between direct and indirect evidence, node-splitting models were applied (20). We tested the consistency of each network using node-splitting analyzes. For each comparison involving direct data, the split node approach assesses whether the effect observed from the direct data aligns with that estimated from indirect-only data.

In the Bayesian approach, the probability of each treatment being ranked best was estimated based on its posterior distribution, and summarized using the surface under the cumulative ranking curve (SUCRA). A higher SUCRA value indicates a superior probability of an intervention (19, 21). Treatment effects were synthesized as standardized mean differences (SMDs) with corresponding 95% credible intervals (CrIs). Statistical significance was defined as a two-sided *p*-value≤0.05 or a 95% CrI not containing the null value (i.e., SMD = 0).

### 2.5 Assessment of potential publication bias

Publication bias was assessed using funnel plots, which graphically display the SMDs and standard error of overall QoL and its individual sub-domains. If there are no publication bias, individual studies are symmetrically distributed at the top of the funnel. If there was a publication bias, they are asymmetrically distributed outside the funnel. In addition, the Egger linear regression test was conducted to detect statistical evidence of publication bias (19, 22, 23).

### 2.6 Quality assessment

The risk of bias and methodological quality was evaluated using the Cochrane Collaboration Risk of Bias tool version 2 (RoB 2) (24). We assessed 5 parameters, including: (1) randomization process, (2) deviations from the intended interventions, (3) missing outcome data, (4) measurement of the outcomes, and (5) selection of the reported results. Each domain was rated as “low risk,” “some concerns,” or “high risk” according to the RoB 2 algorithm. Two reviewers independently assessed each study, and any discrepancies were resolved through discussion and consensus.

### 2.7 Certainty of evidence assessment

The certainty of evidence was assessed using the semi-automated CINeMA (Confidence in network meta-analysis) web application. This tool evaluates the quality of evidence based on six domains—within-study bias, reporting bias, indirectness, imprecision, heterogeneity, and inconsistency—to grade the confidence of estimates as high, moderate, low, or very low (25, 26).

## 3 Results

### 3.1 Study selection and description of included studies

**Figure 1:**
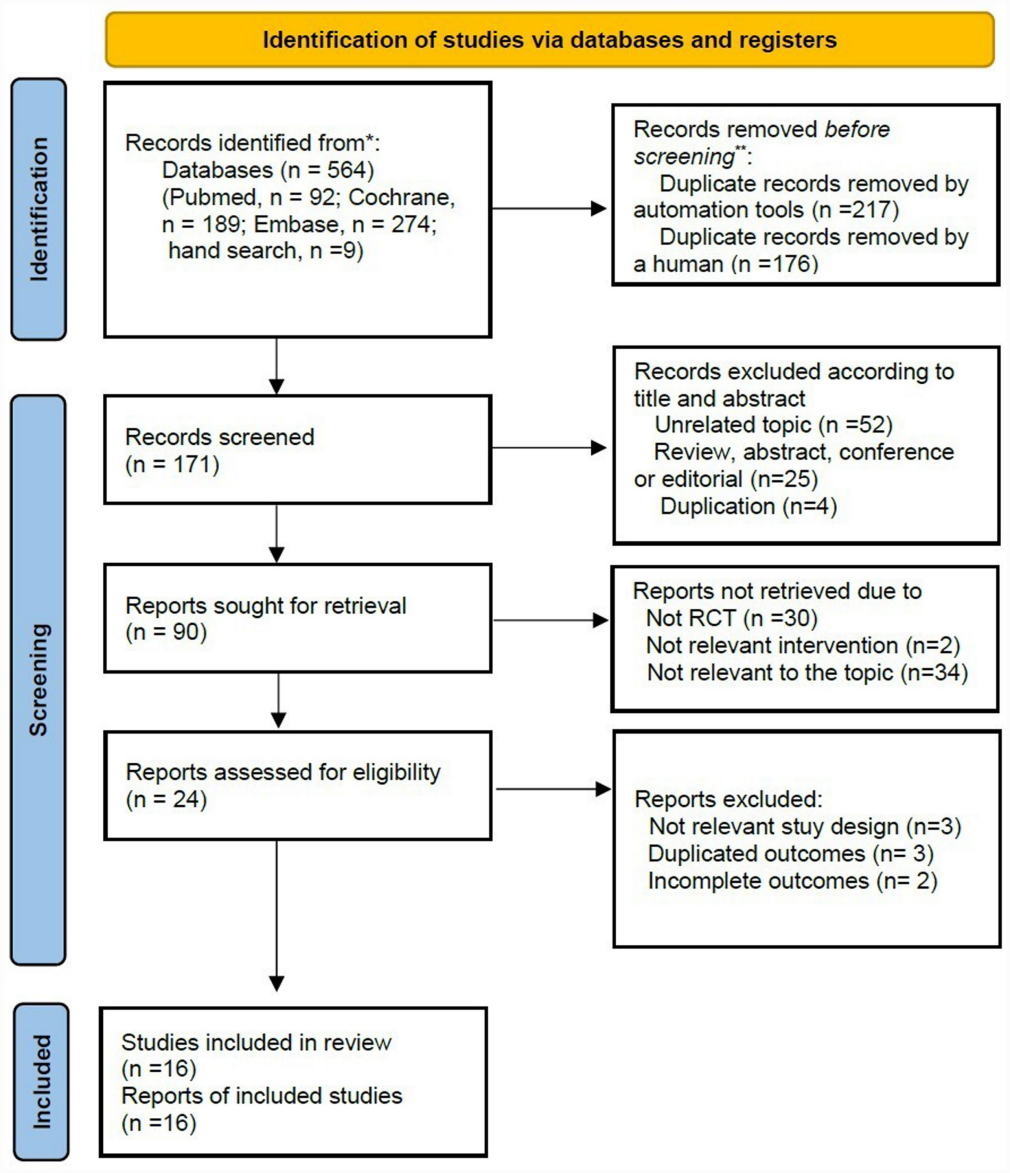
PRISMA flow diagram.

A total of 564 records were identified through database searches (PubMed/Medline, *n* = 92; Cochrane Library, *n* = 189; and Embase, *n* = 274) and additional manual search (*n* = 9). After removing duplicates using automated tools (*n* = 217) and performing manual review (*n* = 176), 171 records remained. Of these, 81 records were excluded based on the title and abstract review. The remaining 90 studies were reviewed, of which 73 were excluded for the following reasons: non-randomized controlled trials (RCTs) (*n* = 30) or unrelated to the interventions or target conditions (*n* = 36). Twenty-four studies underwent full-text reviews. Of these, seven were excluded due to inappropriate study designs (*n* = 3), duplicated reports (*n* = 3), or incomplete outcome data (*n* = 2). Finally, sixteen studies were deemed eligible for qualitative and quantitative synthesis (Figure 1).

The researchers conducted a systematic literature review and NMA of 16 studies involving a total of 3,802 participants. The included RCTs (10, 11, 27–40) were published between 2004 and 2024 and were conducted in various countries. The included trials evaluated MAO-B inhibitors (rasagiline and safinamide), COMT inhibitor (entacapone and opicapone), and the combination of rasagiline and pramipexole (P2B001), all of which compared with placebo or active intervention. Study duration varied from 12 to 26 weeks. Across studies, the mean age of participants ranged from 58.5 to 72.3 years, and the proportion of female participants varied between 27% and 64%. Study populations included individuals with early or advanced PD, with or without motor fluctuations, reflecting a diverse spectrum of clinical disease stages. A detailed summary of study characteristics, including inclusion Criteria and outcome measures, is presented in Table 1.

**Table 1:** Characteristics of included studies in the network meta-analysis.

Study	Country	Study design	Treatment/Intervention	Age (years)	Female (%)	Sample size	Follow-up, (weeks)	Outcome variables	Inclusion criteria
Lee et al. (38)	Korea	RCT	Opicapone 60 mg	64.1 ± 7.5	49 (55.7)	88	4	PDQ-8	PD patients with motor fluctuation
			Levodopa 100 mg	64.2 ± 8.0	37 (45.7)	81			
Olanow et al. (27)	USA	RCT	P2B001 (pramipexoleER0.6 mg + rasagilineER0.75 mg)	63.9 ± 9.4	56 (32.5)	157	12	PDQ-39	Untreated early PD patients
			Pramipexole ER 0.6 mg	64.9 ± 8.4	52 (33.3)	156			
			Rasagiline ER 0.75 mg	65.1 ± 9.5	48 (31.2)	154			
			Prami ER 3.2 mg	63.9 ± 8.8	24 (31.2)	77			
Hattori et al. (28)	Japan	RCT	Placebo	68.64 ± 7.66	79 (58.1)	136	24	PDQ-39	PD patients with motor fluctuation
			Safinamide 100 mg	68.36 ± 9.04	65 (50.8)	128			
			Safinamide 50 mg	67.18 ± 9.04	73 (55.7)	131			
Kulisevsky et al. (36)	Spain	RCT	Placebo	72.3 ± 10	5 (35.7)	14	24	PDQ-39	Non-demented patients with PD
			Safinamide	66.7 ± 9.2	4 (30.82)	13			
Wei et al. (29)	China	RCT	Placebo	68.85 ± 6.24	69 (44.8)	154	16	PDQ-39	PD patients with motor fluctuation
			Safinamide 100 mg	70.43 ± 8.13	59 (39.1)	151			
Hattori et al. (30)	Japan	RCT	Placebo	66.3 ± 7.62	88 (62.4)	141	26	PDQ-39	PD patients with motor fluctuation
			Rasagiline 1 mg	65.8 ± 8.48	83 (64.3)	129			
			Rasagiline 0.5 mg	66.1 ± 8.74	76 (56.7)	134			
Zhang et al. (31)	China	RCT	Placebo	61.7 ± 9.9	49 (31)	158	16	PDQ-39	PD patients with motor fluctuation
			Rasagiline 1 mg	62.7 ± 8.9	60 (37)	163			
Zhang et al. (32)	China	RCT	Placebo	59.5 ± 9.2	25 (38.5)	65	26	PDQ-39	Early PD patients more than 35 years of age and HYSS score less than 3
			Rasagiline	58.5 ± 8.7	30 (46.2)	65			
Lees et al. (37)	White, Asian, other	RCT	Placebo	61.5 ± 8.9	64 (47.4)	135	14–15	PDQ-39	PD patients with motor fluctuation
			Opicapone 25 mg	62.5 ± 8.5	43 (34.4)	125			
			Opicapone 50 mg	65.5 ± 8.4	58 (39.5)	147			
Olanow et al. (33)	USA, Israel	RCT	Placebo	64.5 ± 7.7	19 (38)	50	12	PDQ-39	Untreated early PD patients
			P2B001 (pramipexole ER 0.3 mg + rasagiline ER 0.75 mg)	63.5 ± 8.8	16 (32)	50			
			P2B001 (pramipexole ER 0.6 mg + rasagiline ER 0.75 mg)	62.9 ± 8.1	14 (29)	49			
Schapira et al. (11)	White, Asian	RCT	Placebo	62.1 ± 8.9	112 (40.7)	275	24	PDQ-39	PD patients with motor fluctuation
			Safinamide	61.7 ± 9.0	103 (37.6)	274			
Ferreira et al. (10)	Europe, Russia	RCT	Placebo	64.3 ± 9.3	50 (41)	121	14–15	PDQ-39	PD patients with motor fluctuation
			Entacapone 200 mg	63.7 ± 8.8	46 (38)	122			
			Opicapone 5 mg	63.6 ± 9.3	51 (42)	122			
			Opicapone 25 mg	64.4 ± 9.0	52 (44)	119			
			Opicapone 50 mg	63.5 ± 9.2	46 (40)	115			
Barone et al. (34)	Italy	RCT	Placebo	66.1 ± 8.35	27 (41.5)	65	12	PDQ-39	Non-demented PD patients with depressive symptoms
			Rasagiline 1 mg	66.0 ± 8.74	31 (53.4)	58			
Borgohain et al. (40)	Italy, Romania, India	RCT	Placebo	59.4 ± 9.41	62 (27.9)	222	24	PDQ-39	PD patients with motor fluctuation
			Safinamide 100 mg	60.1 ± 9.19	61 (27.2)	224			
			Safinamide 50 mg	60.1 ± 9.65	66 (29.6)	223			
Reichmann et al. (39)	Europe	RCT	Placebo	66.0 ± 9.0	59 (33.9)	96	13	PDQ-39	PD patients with motor fluctuation
			Entacapone 200 mg	67.0 ± 8.0	39 (40.6)	174			
Olanow et al. (35)	USA	RCT	Placebo	70.2 ± 9.4	118 (31.3)	377	26	PDQ-39	PD patients without motor fluctuation
			Entacapone 200 mg	69.8 ± 9.3	101 (27.1)	373			

ER, Extended Release; PD, Parkinson’s disease; PDQ, Parkinson’s disease questionnaire; RCT, randomized controlled trial.

### 3.2 Findings from network meta-analysis

The assumption of consistency was evaluated using the node-splitting approach. No evidence of inconsistency was observed between direct and indirect comparisons for overall quality of life and activities of daily living (all *p* > 0.05). For the remaining outcomes, statistical assessment of inconsistency was not feasible due to the absence of closed loops within the network.

#### 3.2.1 Overall QoL analysis

**Figure 2:**
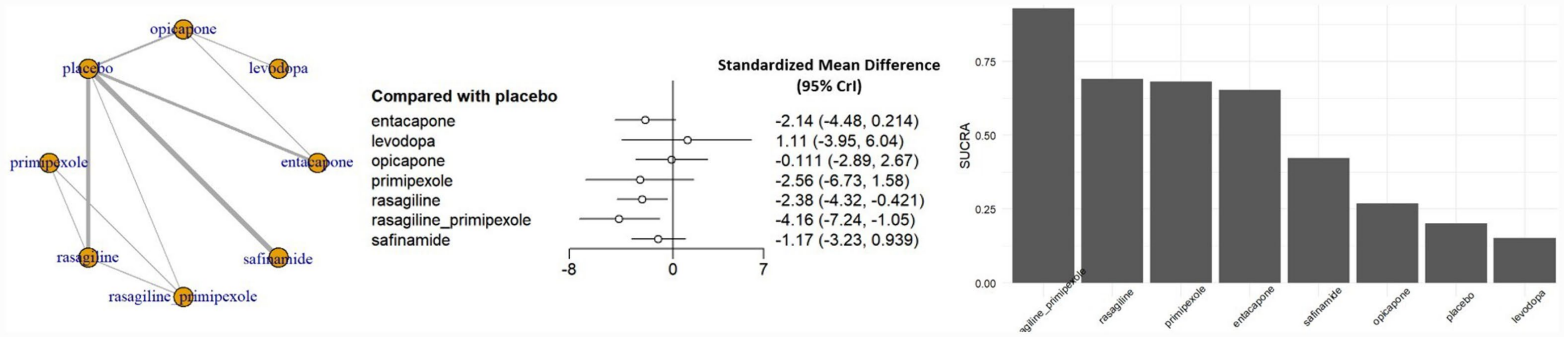
Network meta-analysis results and SUCRA values for overall QoL. CrI, credible interval; SUCRA, surface under the cumulative ranking curve.

The overall QoL analysis was conducted using the standard therapeutic dose for each intervention. The combination of rasagiline ER with pramipexole ER demonstrated the most pronounced improvement in overall QoL compared with placebo (SMD, −4.16; 95% CrI, −7.24 to −1.05), followed by rasagiline monotherapy (SMD, −2.38; 95% CrI, −4.32 to −0.42), both demonstrating statistically significant effects. Safinamide (SMD, −1.17; 95% CrI, −3.23 to 0.94) and entacapone (SMD, −2.14; 95% CrI, −4.48 to 0.21) showed improvements, but were not statistically significant. Neither opicapone nor levodopa demonstrated any appreciable benefits (Figure 2).

For the primary outcome of Overall QoL, the overall certainty of evidence across the network ranged from low to moderate (Supplementary Table S3). A significant proportion of pairwise comparisons were downgraded due to serious imprecision, characterized by 95% confidence intervals (CrI) that were sufficiently wide to encompass effects favoring either intervention. Consequently, although the point estimates often suggested clinically meaningful differences, the true treatment effects remain uncertain. This imprecision is likely attributable to the sparse data resulting from the small number of studies available for each specific comparison.

#### 3.2.2 Subdomain analysis of QoL

In addition to evaluating overall QoL, we performed sub-analyzes by individual PDQ-sub-domain. Significant improvements were identified in a few sub-domains, particularly those related to activities of daily living (ADL) and emotional well-being.

**Figure 3:**
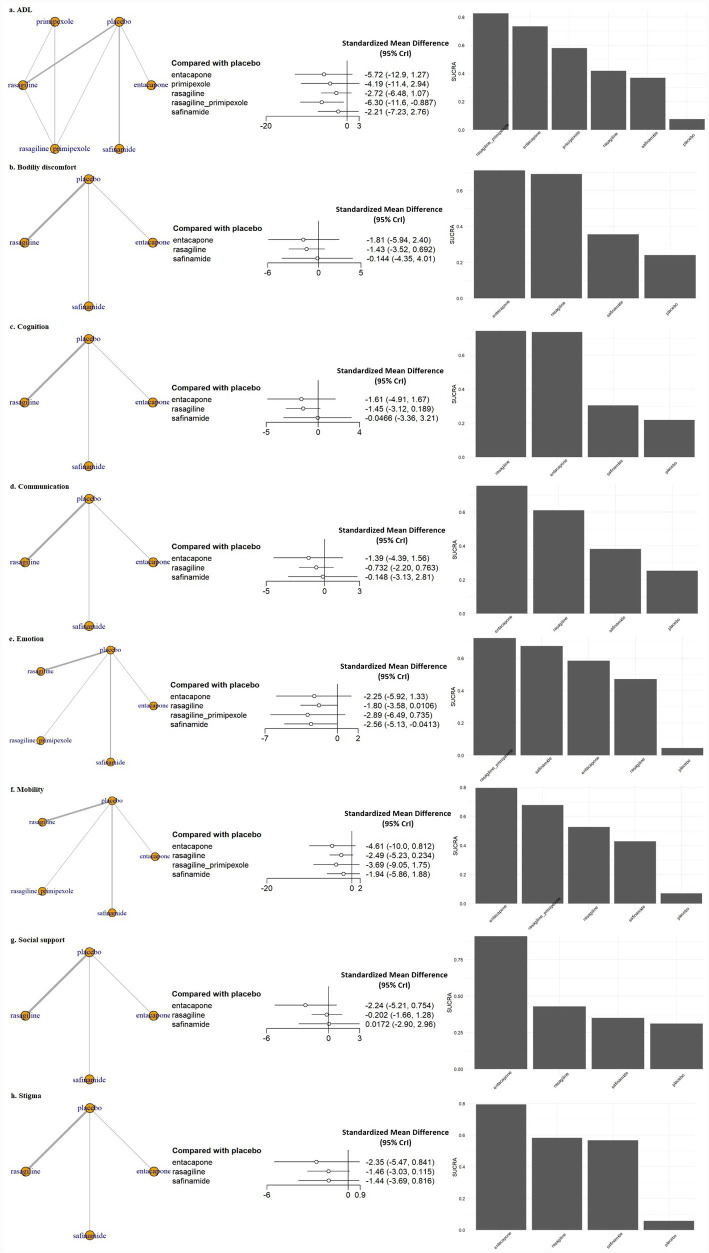
Network meta-analysis results and SUCRA values for subdomains of QoL. ADL, activities of daily living; CrI, credible interval; Emotion, emotional well-being; SUCRA, surface under the cumulative ranking curve.

In the ADL domain, combination therapy with pramipexole ER and rasagiline ER produced a clinically meaningful and statistically significant improvement compared to placebo (SMD, −6.30; 95% CrI, −11.6 to −0.89) (Figure 3). This trend was similarly observed in the dose-stratified analysis (SMD, −6.17; 95% CrI, −14.10 to 1.54), although it was not statistically significant. In contrast, monotherapy with either pramipexole ER or rasagiline ER at the same dose failed to demonstrate significant benefit (Figure 3; Supplementary Figure S1).

In the emotional well-being domain, safinamide 100 mg significantly reduced emotional burden compared with placebo (SMD, −2.56; 95% CrI, −5.13 to −0.04) (Figure 3), a finding that was consistently supported in the dose-stratified analysis (SMD, −2.43; 95% CrI, −4.58 to −0.30) (Supplementary Figure S1). Conversely, safinamide 50 mg did not yield a statistically significant effect (SMD, −1.81; 95% CrI, −4.42 to 0.73), suggesting a possible dose–response relationship. Rasagiline 1 mg monotherapy also showed a trend of improvement in emotional well-being compared with placebo (SMD, −1.81; 95% CrI, −3.42 to −0.19) (Figure 3). No statistically significant differences were observed between active treatments and placebo in other sub-domains, such as bodily discomfort, cognition, communication, mobility, social support and stigma, across both standard dose and dose-stratified analyzes.

#### 3.2.3 Surface under the cumulative ranking curve

Figure 2 presents the surface under the cumulative ranking curve (SUCRA) values for the overall QoL outcome. The combination therapy of rasagiline ER and pramipexole ER ranked the highest (SUCRA = 0.84), followed by rasagiline monotherapy. All evaluated MAO-B and COMT inhibitors had higher SUCRA values than placebo. Figure 3 shows the SUCRA rankings across all QoL sub-domains. In the ADL domain, the combination of rasagiline ER and pramipexole ER had the highest ranking, with entacapone having the second highest SUCRA value, but these treatment effects were not statistically significant. In the emotional well-being domain, the combination of rasagiline ER and pramipexole ER showed the highest SUCRA ranking, although the effect was not statistically significant, while safinamide 100 mg also ranked highly and demonstrated a statistically significant improvement. In all other QoL sub-domains, SUCRA values were generally lower, and no statistically significant differences were observed between active treatments and placebo.

### 3.3 Publication bias assessment

To evaluate the potential for publication bias across the included studies, funnel plots were generated for QoL and each of the QoL sub-domains (Supplementary Figure S2). Visual inspection showed symmetrical distributions around the central line in most outcomes, including overall QoL, activities of daily living (ADL), cognition, and emotional well-being, indicating no evidence of publication bias or small-study effect in this meta-analysis. Quantitative assessments using Egger’s regression test and Begg and Mazumdar’s rank correlation test further supported these observations, with all *p*-values exceeding the threshold for significance (*p* > 0.05 for all outcomes).

### 3.4 Quality assessment

**Figure 4:**
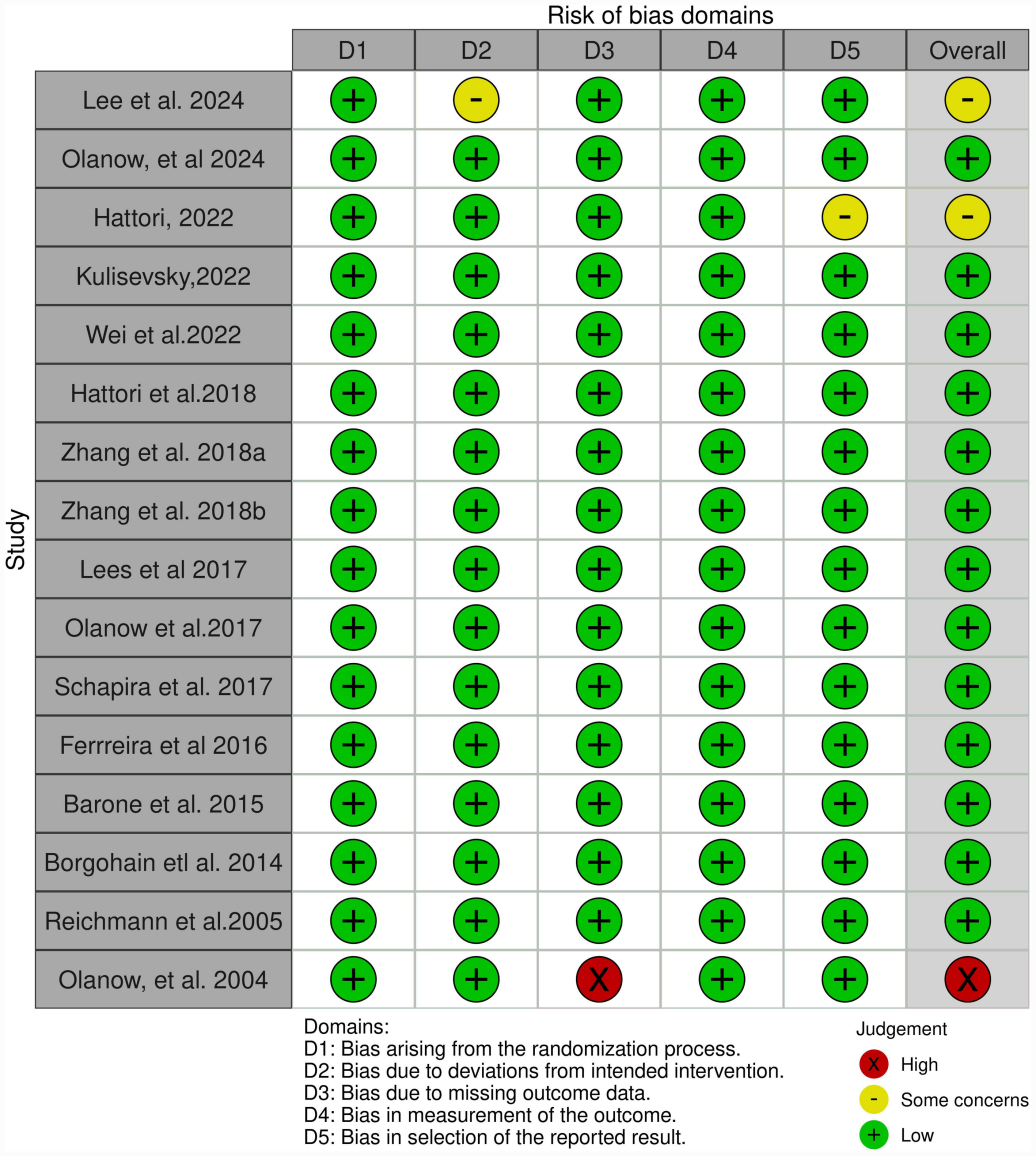
Quality assessment for individual studies.

A total of 16 RCTs were evaluated using the Cochrane RoB 2.0 tool. As shown in Figure 4, most studies were judged to have an overall low risk of bias across the five domains. Specifically, all studies were at “*low*” risk in domains 1 (randomization process), 2 (deviation from intended intervention), and 4 (outcome measurement). In domain 5 (choice of reported outcomes), two studies by Hattori et al. (28) and Lee et al. (38) were rated as having “*some concerns*,” resulting in an overall risk of bias rating of “*some concerns*” for these trials. In Domain 3 (missing outcome data), one study Olanow et al. (35) was rated as a “*high*” risk of bias due to incomplete follow-up or lack of appropriate handling of missing data. This study was also judged to have a “*high*” risk of bias overall. All other studies were considered methodologically robust with a “*low*” risk of bias.

## 4 Discussion

In this NMA, we systematically assessed the impacts of commonly prescribed MAO-B and COMT inhibitors on QoL outcomes in patients with PD. Our primary objective was to determine whether these medications confer meaningful improvements in the overall QoL beyond their established efficacy in ameliorating motor symptoms. Furthermore, recognizing the multi-dimensional aspects of QoL in PD, we conducted sub-domain-specific analyzes to evaluate the influence of these medications across individual QoL sub-domains. This comprehensive approach aimed to enhance our understanding of the potential QoL benefits associated with MAO-B and COMT inhibitors in patients with PD.

In the analysis of overall QOL measures, the combination of rasagiline ER and pramipexole ER was associated with the most pronounced and statistically significant improvement in QoL. Rasagiline monotherapy also showed a significant benefit. While safinamide and entacapone showed a trend toward improvement, the effects were not significant. These findings support the use of rasagiline, particularly in pramipexole ER–containing regimens, as a preferred treatment option for patients with impaired QoL in PD.

Rasagiline is a selective, irreversible MAO-B inhibitor that enhances synaptic dopamine and alleviates motor symptoms in PD (41). Unlike selegiline, it is metabolized into aminoindan, a non-amphetamine compound with potential neuroprotective effects. Additional mechanisms such as NMDA receptor modulation and reduced glutamate excitotoxicity may further support treatment adherence and QoL (42). In the TEMPO study, rasagiline significantly improved PDQUALIF scores, particularly in self-image and emotional well-being (43). More recently, NMAs have also ranked rasagiline higher than selegiline and safinamide in global PDQ-39 scores and key subdomains such as ADL and emotional well-being (44, 45). These findings are aligned with the QoL benefits observed in our study.

In contrast, our study identified the combination of extended-release pramipexole and rasagiline as the most effective treatment for improving QoL, consistent with findings from a RCT by Olanow et al. (33). In that study, a fixed low-dose combination of pramipexole ER (0.6 mg) and rasagiline ER (0.75 mg) significantly improved PDQ-39 total scores and key subdomains, including ADL and emotional well-being, compared with placebo and either monotherapy. The concordance with this prior evidence suggests that the complementary mechanisms of dopamine agonism and MAO-B inhibition may exert synergistic effects on both motor and non-motor symptoms, leading to clinically meaningful improvement in patient-perceived QoL. Nevertheless, this finding warrants cautious interpretation. Because pramipexole is a dopamine receptor agonist, the observed benefits associated with the fixed-dose combination P2B001 may partly reflect additive effects of direct dopamine receptor stimulation, rather than the effect of MAO-B inhibition alone. Accordingly, the results related to P2B001 should be interpreted as hypothesis-generating, highlighting the potential value of combination strategies, rather than providing definitive evidence of class-specific superiority of MAO-B inhibitors. In addition, the magnitude of the observed standardized mean differences should be interpreted cautiously. Relatively large SMD values may partly reflect statistical features of the included trials, such as small sample sizes and low within-study variability. Because SMDs represent relative, variance-adjusted effects, they should not be directly interpreted as absolute changes in QoL scores or as excessively large clinical benefits.

In the analysis of individual QoL sub-domains, the most notable improvements were observed in ADL and emotional well-being. The combination of pramipexole ER and rasagiline ER significantly enhanced ADL scores, consistent with overall QoL outcomes. Additionally, safinamide 100 mg led to a statistically significant reduction in emotional burden, whereas the 50 mg dose did not, suggesting a potential dose–response relationship. These findings are in line with previous studies (11, 40, 46, 47). A *post hoc* analysis of the Study 016 and SETTLE trials found that safinamide 100 mg, but not 50 mg, significantly improved NMSs, particularly mood and sleep disturbances (47). Other studies have also demonstrated that the 100 mg dose of safinamide benefits pain, fatigue, and depressive symptoms in patients with motor fluctuations (28, 46–48). These effects are likely mediated by safinamide’s dual mechanism of action, which includes reversible MAO-B inhibition and modulation of glutamate release through voltage-gated sodium and calcium channels (49). This multimodal pharmacologic profile is thought to play a role in reducing NMS burden, which is a major determinant of QoL in PD. Recent studies have further clarified the role of safinamide in Asian patients with PD. Pooled analyzes of Asian cohorts and dedicated phase III studies suggest that safinamide, when added to levodopa, is associated with reductions in OFF time, improvements in motor function, and favorable changes in selected PD specific QoL measures, without new safety concerns (15). These benefits appear to be maintained in older patients, including those aged 75 years or older. In addition, *post hoc* analyzes of large international trials indicate that the magnitude of clinical benefit and the overall safety profile of safinamide are broadly comparable between Asian and non-Asian populations, although these findings are derived from indirect evidence and should be interpreted with appropriate caution (50).

In our analysis, COMT inhibitors, such as entacapone and opicapone, did not show statistically significant improvements in QoL when compared to MAO-B inhibitors. This is consistent with findings from the PD MED study, which reported greater improvements in mobility and EQ-5D index scores with MAO-B inhibitors than with COMT inhibitors (14). These differences may be explained partly by the pharmacologic characteristics of COMT inhibitors. While these agents act peripherally to prolong the half-life of levodopa and help stabilize motor fluctuations, they do not directly modulate central dopamine levels. Consequently, their effects on NMSs, such as depression, sleep disturbances, or pain, may be relatively limited. Nevertheless, in our individual sub-domain analyzes, entacapone ranked relatively high in SUCRA scores for bodily discomfort, communication, and mobility, suggesting that improvements in motor function may contribute to certain aspects of QoL. Furthermore, recent real-world evidence from the OPEN-PD study demonstrated that opicapone significantly reduced NMS burden (−27.3% in NMSS) and improved QoL (−18.4% in PDQ-39 SI) after 6 months, suggesting that COMT inhibitors, particularly opicapone, may offer clinically meaningful, potentially central or indirect benefits not consistently demonstrated in controlled clinical trials (51).

This study underscores the potential of MAO-B inhibitors, particularly rasagiline and safinamide, to improve QoL in PD, with notable effects in emotional well-being and daily functioning. The combination of rasagiline ER and pramipexole ER (P2B001) showed the greatest benefits, suggesting additive dopaminergic effects. Although COMT inhibitors showed limited impact on overall QoL, they appear to provide improvements in motor-related PDQ-39 or 8 subdomains, particularly mobility and bodily discomfort. These findings support a symptom-guided, individualized approach to adjunctive therapy selection, prioritizing MAO-B inhibitors in patients with significant non-motor symptoms and COMT inhibitors in those with motor fluctuations.

Taken together, the findings of this network meta-analysis support a symptom-guided and individualized approach to adjunctive therapy selection in Parkinson’s disease. MAO-B inhibitors, particularly rasagiline and safinamide, may be considered in patients with levodopa-treated Parkinson’s disease who experience clinically relevant wearing-off accompanied by a substantial non-motor symptom burden or impaired patient-reported quality of life. In contrast, COMT inhibitors may be more appropriate for patients whose primary therapeutic goal is the reduction of OFF time and stabilization of motor fluctuations, particularly when non-motor symptoms are less prominent. These considerations should be interpreted as agent-specific and hypothesis-supporting, rather than definitive treatment recommendations (16).

However, our study has several methodological limitations. First, although most studies used validated QoL instruments such as the PDQ-39 or PDQ-8, inconsistencies in reporting domain-specific outcomes limited detailed sub-domain analyzes. Second, few RCTs prioritized QoL as a primary endpoint, and data were lacking for agents such as selegiline and tolcapone. Uneven study distributions across drug classes limited comprehensive class-wide comparisons. In addition, the present analysis was restricted to standard dosing regimens, primarily due to inconsistent reporting of QoL outcomes across different dose levels, particularly for overall QoL measures. Although limited dose specific data were available for certain QoL sub-domains in a small number of studies, these data were sparse and allowed only exploratory stratified analyzes. As a result, potential dose response relationships could not be systematically evaluated, and the findings should be interpreted within the context of standard dose use in clinical practice. Third, clinical and methodological heterogeneity, such as variations in disease stage, treatment duration, dose, patient characteristics, and baseline QoL, may play some residual confounding roles, even after adjustment using random-effects models. Fourth, Given the progressive course of PD, short follow-up periods (≤ 26 weeks) in most trials limit the assessment of long-term effects on quality of life. These limitations indicate the need for well-designed, head-to-head randomized trials in the future incorporating standardized QoL instruments, longer follow-up periods, and balanced comparisons across drug classes to clarify the domain-specific and sustained impacts of MAO-B and COMT inhibitors on QoL in patients with PD.

## 5 Conclusion

This network meta-analysis highlights the differential impacts of MAO-B and COMT inhibitors on QoL in patients with PD. Rasagiline and safinamide, particularly when used in combination with pramipexole, demonstrated the most consistent benefits across global and non-motor QoL domains, suggesting synergistic effects through dual dopaminergic mechanisms. While COMT inhibitors showed limited effects on overall QoL, agents such as entacapone may contribute meaningfully to motor-related functional domains. These findings support a symptom-guided, individualized approach to treatment selection in PD, emphasizing the differential benefits of MAO-B and COMT inhibitors across non-motor and motor domains.

Importantly, these conclusions are based on evidence derived from a limited number of specific agents within the MAO-B and COMT inhibitor classes and should not be interpreted as class-wide effects. Rather, the findings reflect agent-specific evidence from currently available randomized controlled trials. Further head-to-head randomized trials with standardized quality-of-life instruments and extended follow-up are warranted to confirm these effects and guide optimal treatment strategies.

## Data Availability

Publicly available datasets were analyzed in this study. This data can be found at: all datasets analyzed in this network meta-analysis were obtained from publicly available randomized controlled trials accessible through major bibliographic databases, including PubMed, Embase, and the Cochrane Library. All studies included in the analysis are presented in Table 1. Because the study is based solely on previously published literature, no unique repository or accession number is associated with these datasets.
