# Supplementary material for: Efficacy of MAO-B and COMT inhibitors on quality of life in patients with Parkinson’s disease: a Bayesian network meta-analysis
Source: Front Neurol. 2026 Feb 4;17:1753555. doi: 10.3389/fneur.2026.1753555 (PMC12913192; doi:10.3389/fneur.2026.1753555)
Supplement: Supplementary file 1 [file Data_Sheet_1.pdf]

Supplementary Material

This supplemental material has been provided by the authors to give readers additional information about their work.

S1 Figure. Network meta-analysis Results and SUCRA values for Subdomains of QoL with dose specific
ADL, Activities of Daily Living; CrI, credible interval; Emotion, Emotional well-being; ER, Extend-released; QoL, Quality of Life; SUCRA, Surface Under the Cumulative Ranking Curve

S2 Figure. Funnel plot
Emotion, Emotional well-being; QoL, Quality of Life

S1 Table. Search queries

S2. Table. PRISMA-NMA Checklist: Items to Include When Reporting a Systematic

S3. Table. CINeMA for the primary outcome (QoL)

S1 Figure. Network meta-analysis Results and SUCRA values for Subdomains of QoL with dose specific.

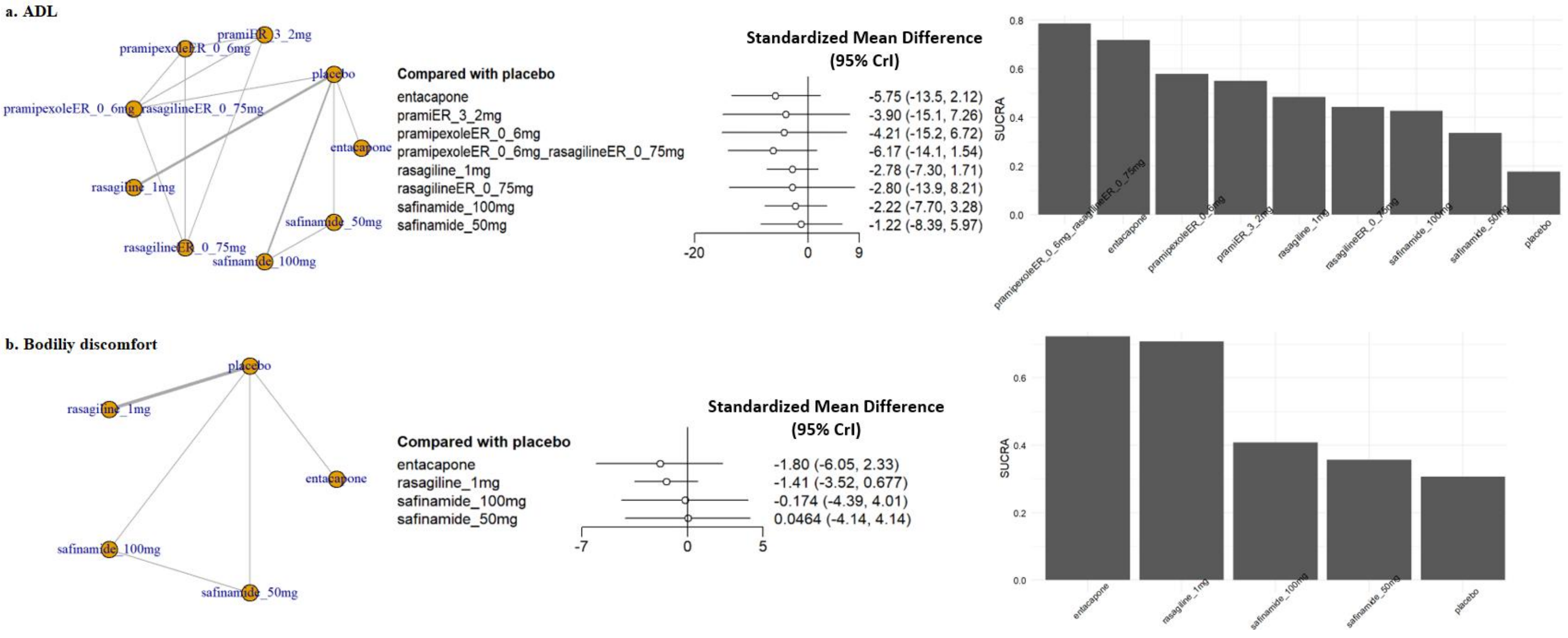

c. Cognition

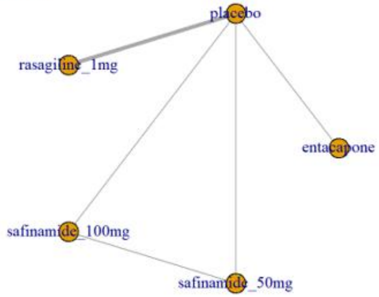

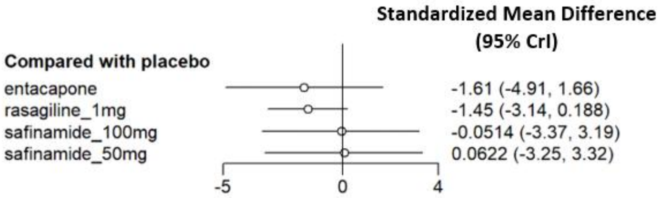

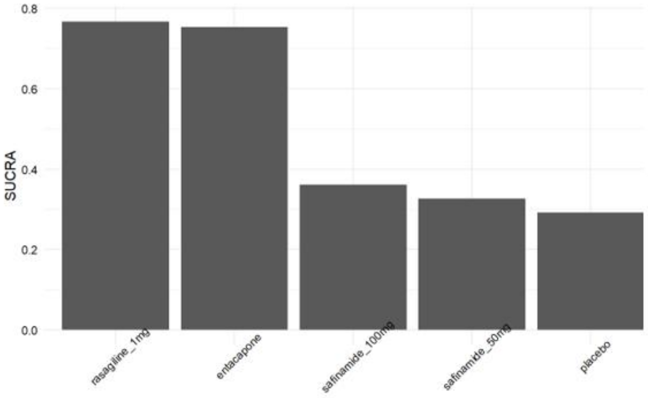

d. Communication

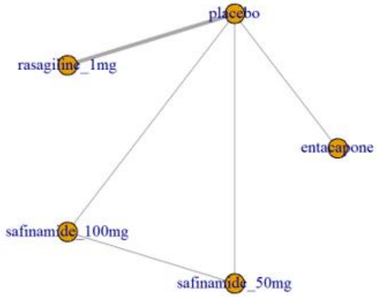

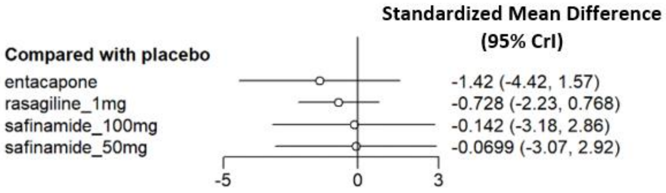

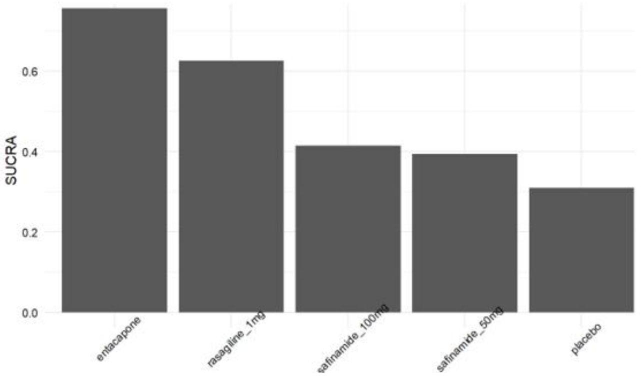

e. Emotion

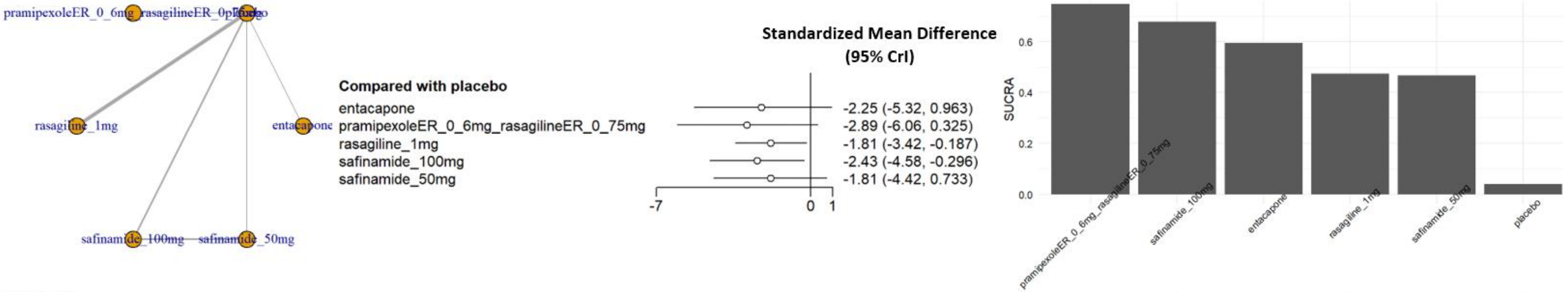

f. Mobility

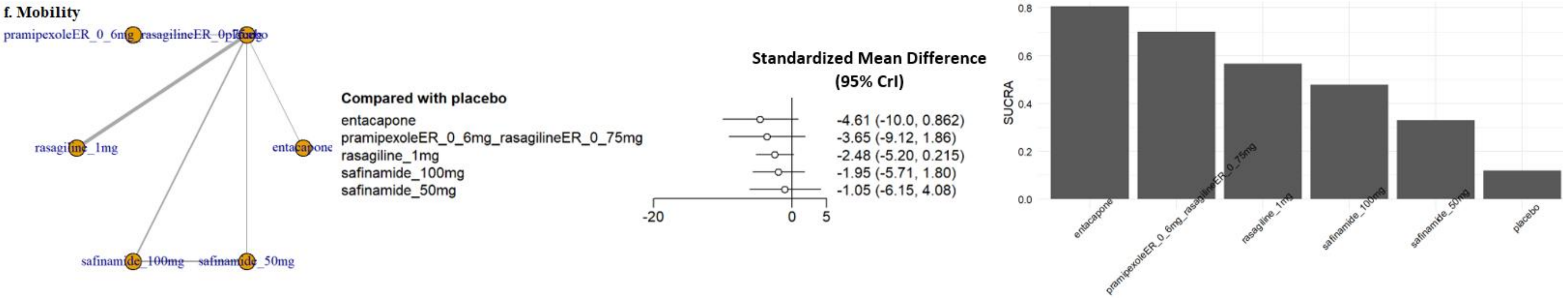

g. Social support

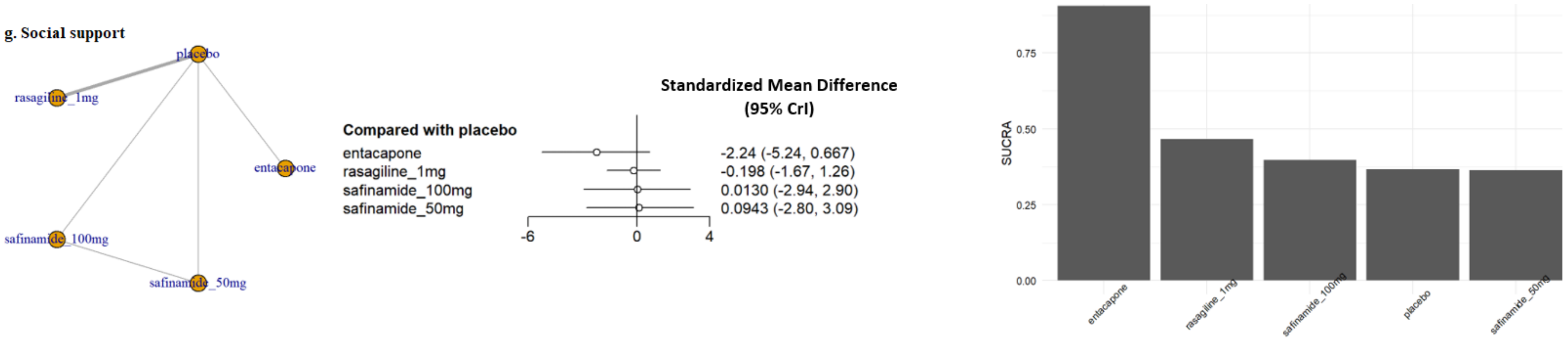

h. Stigma

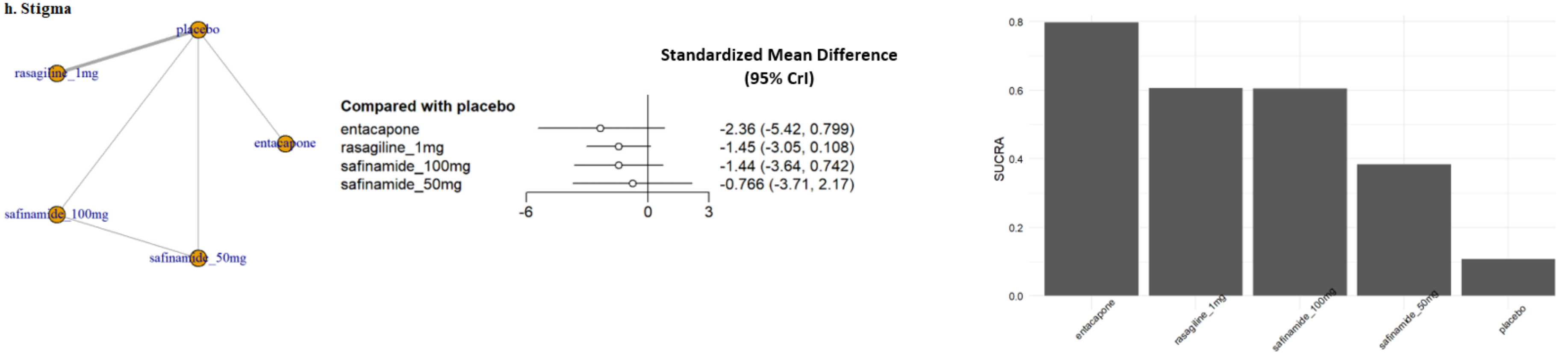

ADL, Activities of Daily Living; CrI, credible interval; Emotion, Emotional well-being; ER, Extend-released; QoL, Quality of Life; SUCRA, Surface Under the Cumulative Ranking Curve

S2 Figure. Funnel plots for publication bias

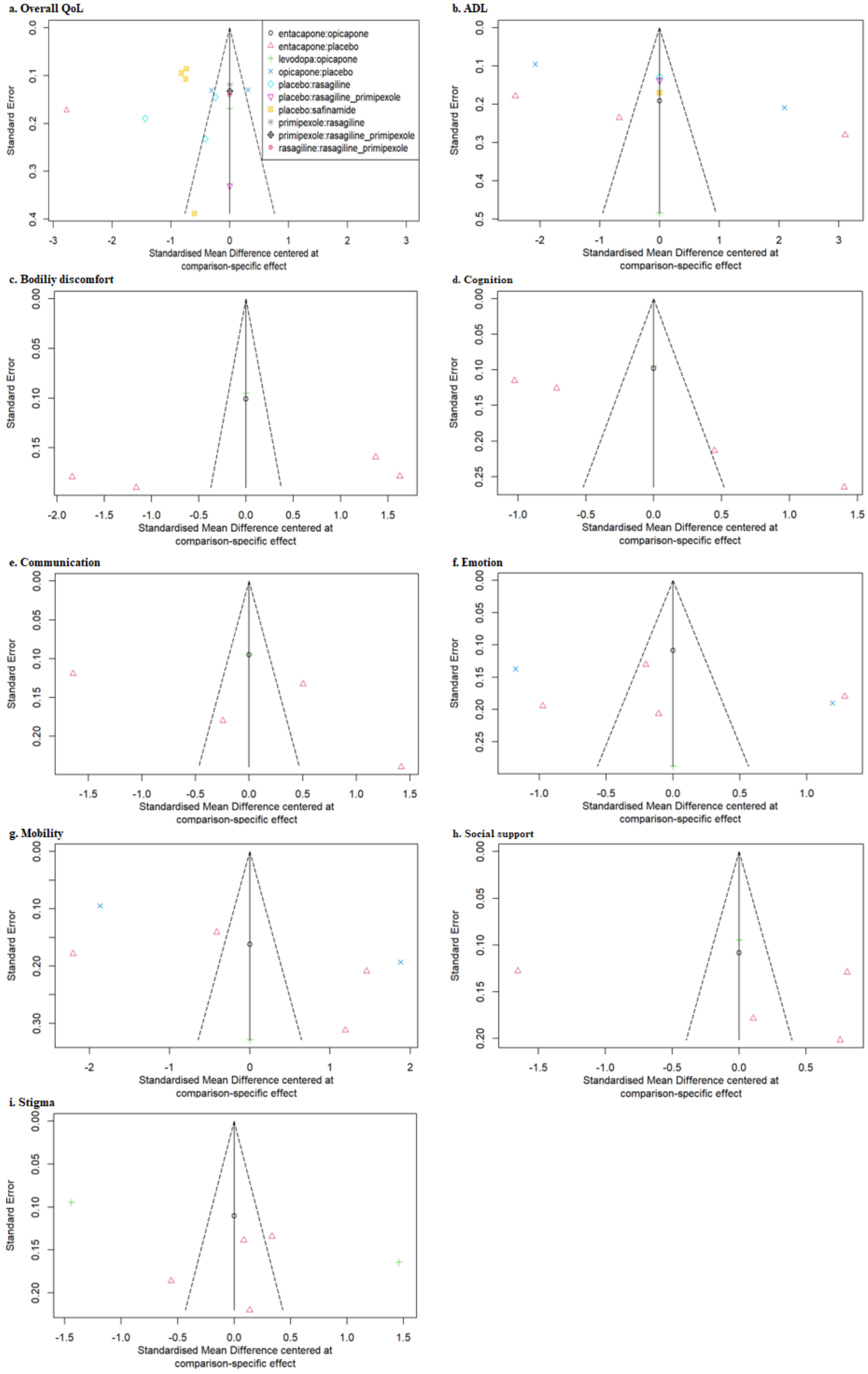

Emotion, Emotional well-being; QoL, Quality of Life

S1 Table. Search queries

PubMed and Cochrane Library

1. Disease
("Parkinson Disease"[Mesh] OR "Parkinson's Disease"[tiab] OR "Parkinson Disease"[tiab])

2. Intervention
("safinamide" [Supplementary Concept] OR "safinamide" [tiab] OR "rasagiline" [Supplementary Concept] OR "rasagiline" [tiab] OR "Selegiline"[Mesh] OR "Selegiline"[tiab] OR "opicapone" [Supplementary Concept] OR "opicapone" [tiab] OR "entacapone" [Supplementary Concept] OR "entacapone" [tiab] OR "Tolcapone"[Mesh] OR "Tolcapone"[tiab])

3. Outcome
("quality of life"[Mesh] OR "quality of life"[tiab] OR "quality of life*"[tiab])

Embase

1. Disease
('parkinson disease'/exp OR ‘Parkinson's Disease’:ti,ab OR ‘Parkinson Disease’:ti,ab)

2. Intervention
('safinamide'/exp OR 'safinamide':ti,ab OR 'rasagiline'/exp OR 'rasagiline':ti,ab OR 'selegiline'/exp OR 'selegiline':ti,ab OR ‘opicapone’/exp OR ‘opicapone’:ti,ab OR ‘entacapone’/exp OR ‘entacapone’:ti,ab OR ‘tolcapone’/exp OR ‘tolcapone’:ti,ab)

3. Outcome
(‘quality of life’/exp OR ‘quality of life’:ti,ab)

S2 Table. **PRISMA NMA Checklist: Items to Include When Reporting a Systematic Review with Network Meta-analysis**

Section/Topic	Item #	Checklist Item	Reported in Section
**TITLE**			
Title	1	Identify the report as a systematic review *incorporating a network meta-analysis (or related form of meta-analysis).*	**Title**
**ABSTRACT**			
Structured summary	2	Provide a structured summary including, as applicable:<br>**Background:** main objectives<br>**Methods:** data sources; study eligibility criteria, participants, and interventions; study appraisal; and *synthesis methods, such as network meta-analysis.*<br>**Results:** number of studies and participants identified; summary estimates with corresponding confidence/credible intervals; *treatment rankings may also be discussed. Authors may choose to summarize pairwise comparisons against a chosen treatment included in their analyses for brevity.*<br>**Discussion/Conclusions:** limitations; conclusions and implications of findings.<br>**Other:** primary source of funding; systematic review registration number with registry name.	**Abstract**
**INTRODUCTION**			
Rationale	3	Describe the rationale for the review in the context of what is already known*, including mention of why a network meta-analysis has been conducted.*_	**Introduction**
Objectives	4	Provide an explicit statement of questions being addressed, with reference to participants, interventions, comparisons, outcomes, and study design (PICOS).	**Introduction**
**METHODS**			
Protocol and registration	5	Indicate whether a review protocol exists and if and where it can be accessed (e.g., Web address); and, if available, provide registration information, including registration number.	**Materials and methods**
Eligibility criteria	6	Specify study characteristics (e.g., PICOS, length of follow-up) and report characteristics (e.g., years considered, language, publication status) used as criteria for eligibility, giving rationale. *Clearly describe eligible treatments included in the treatment network, and note whether any have been clustered or merged into the same node (with justification).*_	Study selection
Information sources	7	Describe all information sources (e.g., databases with dates of coverage, contact with study authors to identify additional studies) in the search and date last searched.	Data sources and literature search
Search	8	Present full electronic search strategy for at least one	Data sources

		database, including any limits used, such that it could be repeated.	and literature search
Study selection	9	State the process for selecting studies (i.e., screening, eligibility, included in systematic review, and, if applicable, included in the meta-analysis).	Study selection
Data collection process	10	Describe method of data extraction from reports (e.g., piloted forms, independently, in duplicate) and any processes for obtaining and confirming data from investigators.	Data extraction
Data items	11	List and define all variables for which data were sought (e.g., PICOS, funding sources) and any assumptions and simplifications made.	Data extraction
**Geometry of the network**	**S1**	Describe methods used to explore the geometry of the treatment network under study and potential biases related to it. This should include how the evidence base has been graphically summarized for presentation, and what characteristics were compiled and used to describe the evidence base to readers.	Network meta-analysis assessment of outcome findings and statistical analysis
Risk of bias within individual studies	12	Describe methods used for assessing risk of bias of individual studies (including specification of whether this was done at the study or outcome level), and how this information is to be used in any data synthesis.	Quality assessment
Summary measures	13	State the principal summary measures (e.g., risk ratio, difference in means). *Also describe the use of additional summary measures assessed, such as treatment rankings and surface under the cumulative ranking curve (SUCRA) values, as well as modified approaches used to present summary findings from meta-analyses.*	Network meta-analysis assessment of outcome findings and statistical analysis
Planned methods of analysis	14	Describe the methods of handling data and combining results of studies for each network meta-analysis. This should include, but not be limited to:<br>• *Handling of multi-arm trials;*<br>• *Selection of variance structure;*<br>• *Selection of prior distributions in Bayesian analyses; and*<br>• *Assessment of model fit.*	Network meta-analysis assessment of outcome findings and statistical analysis
**Assessment of Inconsistency**	**S2**	Describe the statistical methods used to evaluate the agreement of direct and indirect evidence in the treatment network(s) studied. Describe efforts taken to address its presence when found.	Network meta-analysis assessment of outcome findings and statistical analysis
Risk of bias across studies	15	Specify any assessment of risk of bias that may affect the cumulative evidence (e.g., publication bias, selective reporting within studies).	Assessment of potential Publication bias

Additional analyses	16	Describe methods of additional analyses if done, indicating which were pre-specified. This may include, but not be limited to, the following:<br>• Sensitivity or subgroup analyses;<br>• Meta-regression analyses;<br>• *Alternative formulations of the treatment network; and*<br>• *Use of alternative prior distributions for Bayesian analyses (if applicable).*_	Appendix
**RESULTS†**			
Study selection	17	Give numbers of studies screened, assessed for eligibility, and included in the review, with reasons for exclusions at each stage, ideally with a flow diagram.	Study selection and description of included studies
**Presentation of network structure**	**S3**	Provide a network graph of the included studies to enable visualization of the geometry of the treatment network.	Fig2-3
**Summary of network geometry**	**S4**	Provide a brief overview of characteristics of the treatment network. This may include commentary on the abundance of trials and randomized patients for the different interventions and pairwise comparisons in the network, gaps of evidence in the treatment network, and potential biases reflected by the network structure.	Fig2-3
Study characteristics	18	For each study, present characteristics for which data were extracted (e.g., study size, PICOS, follow-up period) and provide the citations.	Table 1
Risk of bias within studies	19	Present data on risk of bias of each study and, if available, any outcome level assessment.	Quality assessment
Results of individual studies	20	For all outcomes considered (benefits or harms), present, for each study: 1) simple summary data for each intervention group, and 2) effect estimates and confidence intervals. *Modified approaches may be needed to deal with information from larger networks.*	Findings from network meta-analysis
Synthesis of results	21	Present results of each meta-analysis done, including confidence/credible intervals. *In larger networks, authors may focus on comparisons versus a particular comparator (e.g. placebo or standard care), with full findings presented in an appendix. League tables and forest plots may be considered to summarize pairwise comparisons.* If additional summary measures were explored (such as treatment rankings), these should also be presented.	Findings from network meta-analysis
**Exploration for inconsistency**	**S5**	Describe results from investigations of inconsistency. This may include such information as measures of model fit to compare consistency and inconsistency models, *P* values from statistical tests, or summary of inconsistency estimates from different parts of the treatment network.	Findings from network meta-analysis
Risk of bias across studies	22	Present results of any assessment of risk of bias across studies for the evidence base being studied.	Publication bias assessment

Results of additional analyses	23	Give results of additional analyses, if done (e.g., sensitivity or subgroup analyses, meta-regression analyses, *alternative network geometries studied, alternative choice of prior distributions for Bayesian analyses,* and so forth).	Appendix
**DISCUSSION**			
Summary of evidence	24	Summarize the main findings, including the strength of evidence for each main outcome; consider their relevance to key groups (e.g., healthcare providers, users, and policy-makers).	**Discussion**
Limitations	25	Discuss limitations at study and outcome level (e.g., risk of bias), and at review level (e.g., incomplete retrieval of identified research, reporting bias). *Comment on the validity of the assumptions, such as transitivity and consistency. Comment on any concerns regarding network geometry (e.g., avoidance of certain comparisons).*	**Discussion**
Conclusions	26	Provide a general interpretation of the results in the context of other evidence, and implications for future research.	**Conclusion**
**FUNDING**			
Funding	27	Describe sources of funding for the systematic review and other support (e.g., supply of data); role of funders for the systematic review. This should also include information regarding whether funding has been received from manufacturers of treatments in the network and/or whether some of the authors are content experts with professional conflicts of interest that could affect use of treatments in the network.	Funding

PICOS = population, intervention, comparators, outcomes, study design.
* Text in italics indicateS wording specific to reporting of network meta-analyses that has been added to guidance from the PRISMA statement.
† Authors may wish to plan for use of appendices to present all relevant information in full detail for items in this section.

***S3 Table.* CINeMA for the primary outcome (QoL)**

Comparison	Number of studies	Within-study bias	Reporting bias	Indirectness	Imprecision	Heterogeneity	Incoherence	Confidence rating	Reason(s) for downgrading
entacapone:opicapone	1	No concerns	Low risk	No concerns	Major concerns	No concerns	No concerns	**Moderate**	["Imprecision"]
entacapone:placebo	3	Some concerns	Low risk	No concerns	No concerns	Major concerns	No concerns	**Low**	["Within-study bias","Heterogeneity"]
levodopa:opicapone	1	Some concerns	Low risk	No concerns	Major concerns	No concerns	No concerns	**Low**	["Within-study bias","Imprecision"]
opicapone:placebo	2	No concerns	Low risk	No concerns	Major concerns	No concerns	No concerns	**Moderate**	["Imprecision"]
placebo:rasagiline	4	No concerns	Low risk	No concerns	No concerns	Major concerns	No concerns	**Moderate**	["Heterogeneity"]
placebo:rasagiline_primipexole	1	No concerns	Low risk	No concerns	No concerns	Major concerns	No concerns	**Moderate**	["Heterogeneity"]
placebo:safinamide	4	No concerns	Low risk	No concerns	Major concerns	No concerns	No concerns	**Moderate**	["Imprecision"]
primipexole:rasagiline	1	No concerns	Low risk	No concerns	Major concerns	No concerns	No concerns	**Moderate**	["Imprecision"]
primipexole:rasagiline_primipexole	1	No concerns	Low risk	No concerns	Major concerns	No concerns	No concerns	**Moderate**	["Imprecision"]
rasagiline:rasagiline_primipexole	1	No concerns	Low risk	No concerns	Major concerns	No concerns	No concerns	**Moderate**	["Imprecision"]
entacapone:levodopa	0	Some concerns	Low risk	No concerns	Major concerns	No concerns	No concerns	**Low**	["Within-study bias","Imprecision"]
entacapone:primipexole	0	No concerns	Low risk	No concerns	Major concerns	No concerns	No concerns	**Moderate**	["Imprecision"]
entacapone:rasagiline	0	No concerns	Low risk	No concerns	Major concerns	No concerns	No concerns	**Moderate**	["Imprecision"]
entacapone:rasagiline_primipexole	0	No concerns	Low risk	No concerns	Major concerns	No concerns	No concerns	**Moderate**	["Imprecision"]
entacapone:safinamide	0	No concerns	Low risk	No concerns	Major concerns	No concerns	No concerns	**Moderate**	["Imprecision"]
levodopa:placebo	0	Some concerns	Low risk	No concerns	Major concerns	No concerns	No concerns	**Low**	["Within-study bias","Imprecision"]
levodopa:primipexole	0	No concerns	Low risk	No concerns	Major concerns	No concerns	No concerns	**Moderate**	["Imprecision"]
levodopa:rasagiline	0	No concerns	Low risk	No concerns	Major concerns	No concerns	No concerns	**Moderate**	["Imprecision"]
levodopa:rasagiline_primipexole	0	No concerns	Low risk	No concerns	No concerns	Major concerns	No concerns	**Moderate**	["Heterogeneity"]
levodopa:safinamide	0	No concerns	Low risk	No concerns	Major concerns	No concerns	No concerns	**Moderate**	["Imprecision"]
opicapone:primipexole	0	No concerns	Low risk	No concerns	Major concerns	No concerns	No concerns	**Moderate**	["Imprecision"]
opicapone:rasagiline	0	No concerns	Low risk	No concerns	Major concerns	No concerns	No concerns	**Moderate**	["Imprecision"]
opicapone:rasagiline_primipexole	0	No concerns	Low risk	No concerns	No concerns	Major concerns	No concerns	**Moderate**	["Heterogeneity"]
opicapone:safinamide	0	No concerns	Low risk	No concerns	Major concerns	No concerns	No concerns	**Moderate**	["Imprecision"]
placebo:primipexole	0	No concerns	Low risk	No concerns	Major concerns	No concerns	No concerns	**Moderate**	["Imprecision"]
primipexole:safinamide	0	No concerns	Low risk	No concerns	Major concerns	No concerns	No concerns	**Moderate**	["Imprecision"]
rasagiline:safinamide	0	No concerns	Low risk	No concerns	Major concerns	No concerns	No concerns	**Moderate**	["Imprecision"]
rasagiline_primipexole:safinamide	0	No concerns	Low risk	No concerns	No concerns	Major concerns	No concerns	**Moderate**	["Heterogeneity"]
